# Urinary Metabolomics on the Biochemical Profiles in Diet-Induced Hyperlipidemia Rat Using Ultraperformance Liquid Chromatography Coupled with Quadrupole Time-of-Flight SYNAPT High-Definition Mass Spectrometry

**DOI:** 10.1155/2014/184162

**Published:** 2014-03-18

**Authors:** Hua Miao, Hua Chen, Xu Zhang, Lu Yin, Dan-Qian Chen, Xian-Long Cheng, Xu Bai, Feng Wei

**Affiliations:** ^1^Department of Physical Education, Northwest University, No. 229 Taibai North Road, Xi'an, Shaanxi 710069, China; ^2^Department of Traditional Chinese Medicine, The College of Life Sciences, Northwest University, No. 229 Taibai North Road, Xi'an, Shaanxi 710069, China; ^3^National Institutes for Food and Drug Control, State Food and Drug Administration, 2 Tiantan Xili, Beijing 100050, China; ^4^Solution Centre, Waters Technologies (Shanghai) Ltd., No. 1000 Jinhai Road, Shanghai 201203, China

## Abstract

Ultraperformance liquid chromatography coupled with quadrupole time-of-flight synapt high-definition mass spectrometry metabolomics was used to characterize the urinary metabolic profiling of diet-induced hyperlipidaemia in a rat model. Analysis was done by orthogonal partial least squares discriminant analysis, correlation analysis, heat map analysis, and KEGG pathways analysis. Potential biomarkers were chosen by S-plot and were identified by accurate mass, isotopic pattern, and MS/MS fragments information. Significant differences in fatty acid, amino acid, nucleoside, and bile acid were observed, indicating the perturbations of fatty acid, amino acid, nucleoside, and bile acid metabolisms in diet-induced hyperlipidaemia rats. This study provides further insight into the metabolic profiling across a wide range of biochemical pathways in response to diet-induced hyperlipidaemia.

## 1. Introduction

Metabolomics is the quantitative measurement of the dynamic multiparametric metabolic responses of living systems to pathophysiological stimuli or genetic modifications [1]. Metabolomics is based on the determination of global metabolite profiles in biological fluids and tissues with subsequent data analysis via a range of multivariate statistical approaches [2]. As a powerful analytical platform, the application of metabolomics has dramatically increased in the fields of physiological evaluation, disease diagnosis, disease prognosis, therapy, biomarker discovery, drug therapy monitoring, and safety and toxicity evaluation [3].

Hyperlipidaemia, as a major risk factor of coronary heart disease, is one of the most important public health problems, with increasing rates of incidence and prevalence [4]. Hyperlipidaemia is defined as a disorder of lipid metabolism leading to abnormal increase of triglycerides (TG), total cholesterol (TC), low-density lipoprotein cholesterol (LDL-C), very low-density lipoprotein cholesterol (VLDL-C), and decrease of high-density lipoprotein cholesterol (HDL-C) [5]. As a progressive chronic and metabolic disease, cardiovascular disease begins in adult and progresses to morbidity and mortality throughout the lifespan. Hyperlipidaemia has an important effect on development and progression of various cardiovascular diseases and atherosclerosis. Both moderate hyperlipidaemia and severe hyperlipidaemia are associated with cardiovascular disease [6]. Recent study indicates that a fundamental defect is an overproduction of large VLDL-C, which triggers a sequence of lipoprotein changes, leading to increased remnant particles, smaller LDL-C, and decreased HDL-C [7]. LDL-C is the primary target for the lipid-lowering therapy and cardiovascular diseases prevention.

Mass spectrometry (MS) and proton nuclear magnetic resonance (^1^H NMR) spectroscopy are two analytical tools commonly used in metabolomics. Recently, an increasing number of ^1^H NMR and gas chromatography-MS (GC-MS) based on metabolomics have been conducted to characterize hyperlipidaemia models and to assess drug treatment [8–12].

Proteomic profiling from insulin resistance and metabolic dyslipidemia rats demonstrated hepatic ER proteins ERp29, ERp46, and ER60; TAP1 and glutamate dehydrogenase were downregulated, whereas P-glycoprotein, *α*-glucosidase, protein disulfide isomerase, fibrinogen, GRP94, and apolipoprotein E were upregulated in the hepatic ER of the fructose-fed hamster [13]. ^1^H NMR-based metabolomics indicated that major metabolic processes like Krebs cycle, cholesterol metabolism, and osmoregulation are perturbed in hypercholesterolaemic rats [9]. GC-MS based on metabolomics showed amino acids (alanine, valine, aspartic acid, phenylalanine, etc.), fatty acids (octadecadienoic acid, arachidonic acid, etc.), and propanoic acid, and glucose and cholesterol were identified as biomarkers in diet-induced hyperlipidaemia rats [12]. Among the analytical techniques in metabolomics research, liquid chromatography is recognized as one of the best analytical techniques in selectivity, sensitivity, and reproducibility [14]. Furthermore, among the various liquid chromatography platforms, ultraperformance liquid chromatography (UPLC) is considered to be suitable for metabolite profiling and metabolomics study, especially for large-scale untargeted metabolic profiling due to its enhanced reproducibility of retention time. UPLC operates with sub-2 *μ*m chromatographic particles and a fluid system capable of operating at pressures up to 15000 psi, providing an increased chromatographic resolution compared to conventional high performance liquid chromatography (HPLC) using larger particles.

In 2005, Wrona et al. introduced the mass spectrometry^ElevatedEnergy^ (MS^E^) data collection technique [15], in which two scanning functions are simultaneously used for data collection. In other words, MS^E^ can provide parallel alternating scans for acquisition at either low collision energy to obtain precursor ion information or high collision energy to obtain full-scan accurate mass fragment, precursor ion, and neutral loss information [14]. MS^E^ involves a simultaneous acquisition through alternating between high and low collision energies during a single chromatographic run. This ability is of major importance, as it offers the structural information required for the identification of unknown biomarkers in the context of untargeted analyses. Recently, the novel quadrupole time-of-flight mass spectrometry with MS^E^ technique has been proven to be a powerful and reliable analytical approach for metabolite identification [16–20]. UPLC Q-TOF/HDMS with MS^E^ technique is becoming increasingly popular in the analysis of biological fluids in the field of metabolomics because it provides high resolution, accurate mass measurement, and structural information [14, 16–20]. A method using UPLC Q-TOF/HDMS/MS^E^-based metabolomics combined with multivariate statistical analysis was applied to rapidly identify urinary metabolite profiling of diet-induced hyperlipidaemia in a rat model. Orthogonal partial least squares discriminant analysis (OPLS-DA), correlation analysis, heat map analysis, and KEGG pathways analysis were performed for investigating the metabolic changes of diet-induced hyperlipidaemia and control rats, and the potential biomarkers were identified accordingly.

## 2. Materials and Methods

### 2.1. Animals and Sample Collection

The study was conducted in accordance with the Regulations of Experimental Animal Administration issued by the State Committee of Science and Technology of People's Republic of China. All procedures and the care of the rats were in accordance with institutional guidelines for animal use in research. Male Sprague-Dawley rats were obtained from the Central Animal Breeding House of Fourth Military Medical University (Xi'an, China). They were maintained at a constant humidity (ca. 60%) and temperature (ca. 23°C) with a light/dark cycle of 12 h. Male rats underwent an adaptation period of several days, during which they were fed a commercial feed. After that they were separated randomly into two groups (*n* = 8/group). Rats were randomly assigned into a diet-induced hyperlipidaemia group and control group. The control group was fed with the common diet during the whole experimental period, and the diet-induced hyperlipidaemia group was fed with high fat diets including 81% basic diet, 10% yolk powder, 7.5% lards, 0.3% sodium cholate, 0.2% methylthiouracil, and 1% cholesterol for continuous 6 weeks. After 6 weeks, individual rats were placed in metabolic cages (1 per cage) to obtain 24-hour urine collections. When urine samples were collected, rats were only freely accessible to water. All the samples were stored at −80°C before analysis.

### 2.2. Sample Preparation

Prior to analysis, urine samples were thawed at room temperature and then centrifuged at 13000 rpm for 10 min to remove solid materials. The supernatant was diluted at a ratio of 3 : 1 with distilled water, mixed, and centrifuged for UPLC analysis.

### 2.3. Chromatographic Separation

The UPLC analysis was performed on a Waters ACQUITY Ultra Performance LC system (Waters, USA) equipped with a Waters Xevo G2 QTof MS. Chromatographic separation was carried out at 45°C on an ACQUITY UPLC HSS T3 column (2.1 mm × 100 mm, 1.8 *μ*m). The mobile phase consisted of water (A) and acetonitrile (B), each containing 0.1% formic acid. The optimized UPLC elution conditions were 0–0.5 min, 1% B; 0.5–12.0 min, 1–30% B; 12.0–15.0 min, 30–99% B; 15.0–16.0 min, 99% B; 16.0–20.0 min, 99.0–1.0% B. The flow rate was 0.45 mL/min. The autosampler was maintained at 4°C. Every 2 *μ*L sample solution was injected for each run.

### 2.4. Mass Spectrometry

Mass spectrometry was performed on a quadrupole and orthogonal acceleration time-of-flight tandem mass spectrometer. The scan range was from 50 to 1200 *m*/*z*. For both positive and negative electrospray modes, the capillary and cone voltage were set at 2.5 kV and 45 V, respectively. The desolvation gas was set at 900 L/h at a temperature of 550°C; the cone gas was set at 50 L/h; the source temperature was set at 120°C. The mass spectrometry was operated in W optics mode with 12,000 resolution using dynamic range extension. The data acquisition rate was set to 0.1 s, with a 0.1 s interscan delay. All analyses were acquired using the LockSpray to ensure accuracy and reproducibility. Leucine-enkephalin was used as the lockmass at a concentration of 300 ng/mL and flow rate of 5 *μ*L/min. Data were collected in centroid mode, the LockSpray frequency was set at 10 s, and data were averaged over 10 scans. All the acquisition and analysis of data were operated by Waters MassLynx v4.1 software.

### 2.5. Analytical Method Assessment

The precision and repeatability of this experiment were tested for assessment of the developed UPLC-MS method according to different chemical polarities and *m*/*z* values; 8 ions including *m*/*z* 284.2934, 340.1060, 282.2779, 256.2620, 367.1490, 296.2360, 372.2366, and 330.0618 were extracted for the assessment according to the variation of their peak areas and retention times. The six parallel random samples were injected to evaluate the sample preparation repeatability. Sample of quality control (QC) was injected. There were six control rats and six diet-induced hyperlipidemia rats; six batches of data from one QC sample could be obtained to evaluate the stability of the UPLC-MS system for the large-scale sample analysis.

### 2.6. Data Analysis

The raw data were analyzed using the MarkerLynx XS software. This software allowed deconvolution, alignment, and data reduction to give a list of mass and retention time pairs with corresponding intensities for all the detected peaks from each data file in the data set. The main parameters were set as follows: retention time range 1–18 min, mass range 50–1000 amu, minimum intensity 1%, mass tolerance 0.01, retention time window 0.20, mass window 0.05, marker intensity threshold 500, and noise elimination level 6. All of the data were normalized to the summed total ion intensity per chromatogram, and the resultant data matrices were introduced to the EZinfo 2.0 software for OPLS-DA. Metabolite peaks were assigned by MS^E^ analysis or interpreted with available biochemical databases, such as HMD, ChemSpider, and KEGG. Potential markers were extracted from S-plots. Correlation analysis and heat maps were analyzed by MetaboAnalyst software. The other statistical analysis was performed by SPSS 11.0. The significant differences between control group and diet-induced hyperlipidemia group were assessed by analysis of variance (ANOVA) followed by *t*-test for multiple comparisons. When comparing with the control group, *P* values less than 0.05 were considered significant.

## 3. Results and Discussion

### 3.1. Sample Preparation and UPLC-MS Analysis

Urine is a complex sample containing various endogenous and exogenous acidic, basic, and neutral compounds with high polarity. Sample preparation by conventional methods including solid-phase extraction or liquid-liquid extraction may lead to loss of high polarity and high hydrophilicity metabolites. While metabolomics is an untargeted analysis of the global changes in endogenous metabolites, conventional methods may cause loss of potential biomarker. Therefore, minimal sample preparation steps were performed on urinary samples in order to avoid the loss of the endogenous metabolites. Urinary samples were centrifuged and diluted prior to the direct injection into UPLC-MS.

**Figure 1:**
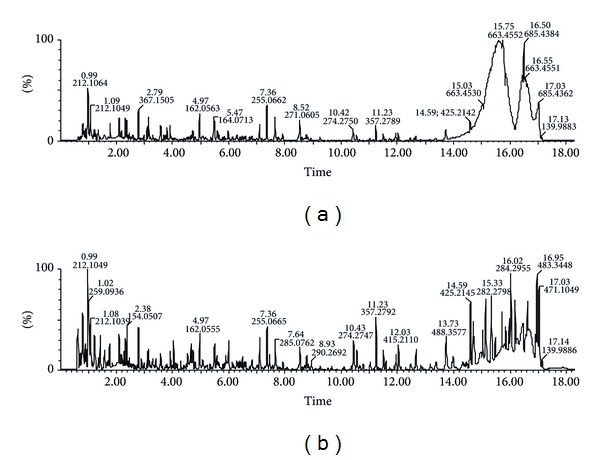
Base peak intensity (BPI) chromatograms obtained from the positive ion UPLC-MS analyses of control (a) and diet-induced hyperlipidemia (b) rats.

The complexity of the urinary sample makes the separation very difficult and consequently results in severe ion suppression. UPLC employs sub-2 *μ*m particle size column, which generates high efficiency to the compound separation and concurrently increases sensitivity and resolution. Thus, UPLC was applied to urinary metabolomics of diet-induced hyperlipidaemia rats in the positive and negative ESI modes. Representative base peak intensity (BPI) chromatogram of the urine of diet-induced hyperlipidaemia rats in the positive ESI mode is shown in Figure 1. Reproducibility was determined from six replicated analyses of the same urinary sample. The variations of *m*/*z* values and retention times of selected peaks in positive ESI mode were less than 8 mDa and 0.05 min, respectively, and the relative standard deviations of peak area and retention time are below 2.9% and 0.78%, respectively. These results indicated the excellent reproducibility and stability during the whole sequence.

### 3.2. Multivariate Data Analysis

**Figure 2:**
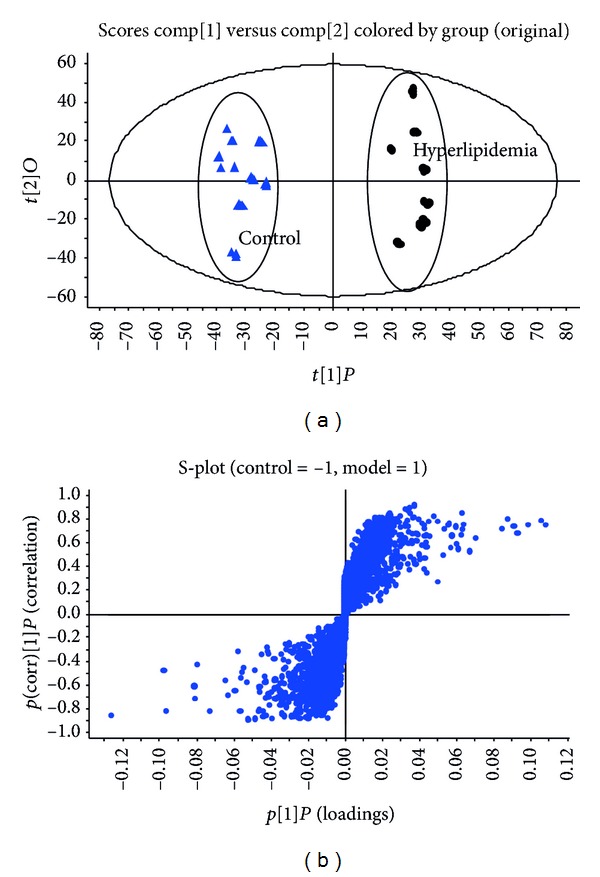
(a) OPLS-DA score plot based on the urinary metabolic profiling of the hyperlipidemia (●) and control (▲) rats; the results indicated that the urinary metabolic pattern was significantly changed in the diet-induced hyperlipidemia rats. (b) S-plot used in our biomarkers selection. The variables marked (□) are the metabolites selected as potential biomarkers. The significant metabolites were selected as potential biomarkers from S-plot and these urinary metabolites are associated with diet-induced hyperlipidemia.

Metabolomics aims at the comparison of samples from a control group and from a case group. The OPLS-DA is an extension of the partial least squares discriminant analysis which integrates an orthogonal signal correction filter to distinguish variations that are useful for prediction of a quantitative response from variations that are orthogonal to prediction. OPLS-DA was demonstrated as a powerful tool for the analysis of qualitative data structures. OPLS-DA score plot was performed on the urinary metabolites from diet-induced hyperlipidemia rats and control rats. According to UPLC-MS data, 8642 peaks of positive ions were detected and processed by MarkerLynx using the same acquiring method.  Figure 2(a) shows the OPLS-DA score plot of the diet-induced hyperlipidemia rats and control rats. The OPLS-DA score plot revealed good fitness and high predictability of the OPLS-DA model with high statistical values of *R* ^2^ and *Q* ^2^. The *R* ^2^ and *Q* ^2^ values are 0.975 and 0.933, respectively. S-plot is a tool for visualization and interpretation of multivariate classification models. It images both the covariance and correlation between the metabolites and the modeled class designation for identification of statistically significant and biochemically interesting metabolites which can avert the increase of false positives (type I error). The significant metabolites were selected as potential biomarkers from S-plot (Figure 2(b)). These urinary metabolites are associated with diet-induced hyperlipidemia.

### 3.3. Identification of Potential Biomarkers

For metabolomics research, the biggest challenge is the identification of potential biomarkers obtained from comparative samples, particularly when they are novel and published work on the compound class is unavailable or prior information is lacking otherwise. All the detected ions were arranged in descending order according to VIP (Variable Importance in the Projection) values, which reflect the influence of each metabolite in control rats and diet-induced hyperlipidemia rats. The more the variable deviates from the origin, the higher the VIP value will be obtained. According to the result of S-plot and reported methods, 16 variables were predicted by comparing the accurate MS and MS^E^ fragments with the metabolites searching in ChemSpider, HMDB, and KEGG, and according to the possible fragment mechanisms, compounds without the given mass fragment information were removed from the candidate list and only the most probable compounds were reserved [21, 22]. MassLynx i-FIT algorithm is used to screen suggested elemental compositions by the likelihood that the isotopic pattern of the elemental composition matches a cluster of peaks in the spectrum, increasing confidence in identified compounds and simplifying results. The lower the i-FIT value, the better the fit [23]. By comparing the retention times and mass spectra to the authentic chemicals, 16 compounds were tentatively identified and shown in Table 1.

### 3.4. Study of Metabolic Changes and Biochemical Interpretation in Diet-Induced Hyperlipidemia Rats

Differences in the levels of metabolites between diet-induced hyperlipidemia rats and control rats were compared using OPLS-DA. The metabolic profiling and multivariate pattern recognition may observe a wider range of metabolites. Since metabolites can be regulated through a number of metabolic pathways, an investigation of the overall features rather than of several selected metabolites enabled us to understand the underlying pathophysiological status more comprehensively.

**Figure 3:**
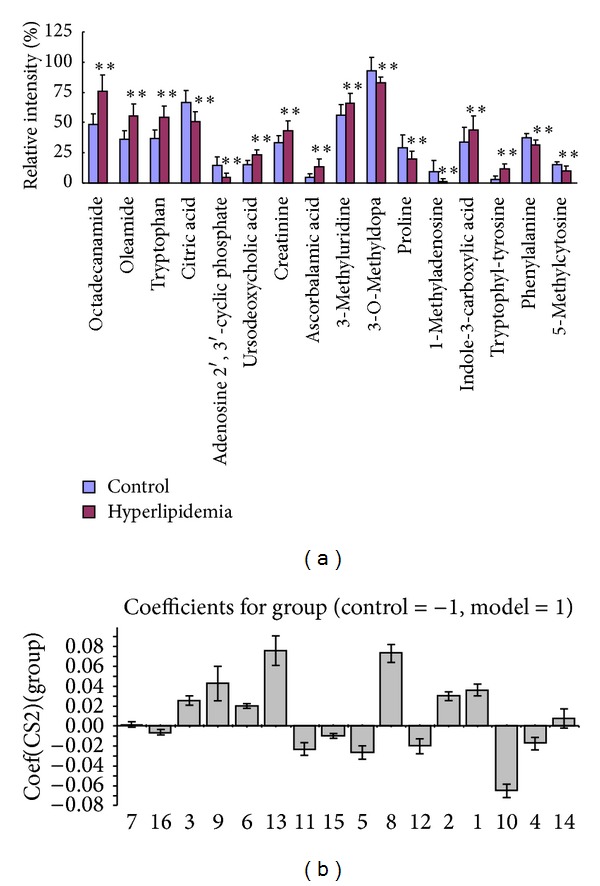
(a) Comparison of the relative intensity and (b) OPLS-DA loading plot of putative potential biomarkers in control and diet-induced hyperlipidaemia rats. The loading plots represent which metabolites are quantitatively higher or lower in diet-induced hyperlipidaemia rats compared with control rats. Numbers consist with Table 1.

To investigate the change of identified metabolites in diet-induced hyperlipidemia rats, relative intensity of identified metabolites compared diet-induced hyperlipidemia rats with control rats. Increased urinary octadecanamide, oleamide, tryptophan, ursodeoxycholic acid, creatinine, ascorbalamic acid, 3-methyluridine, indole-3-carboxylic acid, and tryptophyl-tyrosine and decreased urinary citric acid, adenosine 2′,3′-cyclic phosphate, 3-O-methyldopa, proline, 1-methyladenosine, phenylalanine, and 5-methylcytosine were observed in diet-induced hyperlipidemia rats (Figure 3(a)). Above-mentioned metabolites might play important roles in the metabolic changes of diet-induced hyperlipidemia rats. OPLS-DA loading plot was also generated in diet-induced hyperlipidemia rats and control rats. The loading plot indicated that identified metabolites were quantitatively higher or lower in diet-induced hyperlipidemia rats compared with control rats. Concentrations of identified metabolites including octadecanamide, oleamide, tryptophan, ursodeoxycholic acid, creatinine, ascorbalamic acid, 3-methyluridine, indole-3-carboxylic acid, and tryptophyl-tyrosine were significantly increased in diet-induced hyperlipidemia rats, whereas the concentrations of citric acid, adenosine 2′,3′-cyclic phosphate, 3-O-methyldopa, proline, 1-methyladenosine, phenylalanine, and 5-methylcytosine were decreased in diet-induced hyperlipidemia rats (Figure 3(a)), which are in agreement with the changes of relative intensity (Figure 3(b)). The results demonstrated that these upregulated or downregulated metabolites are associated with diet-induced hyperlipidemia.

**Figure 4:**
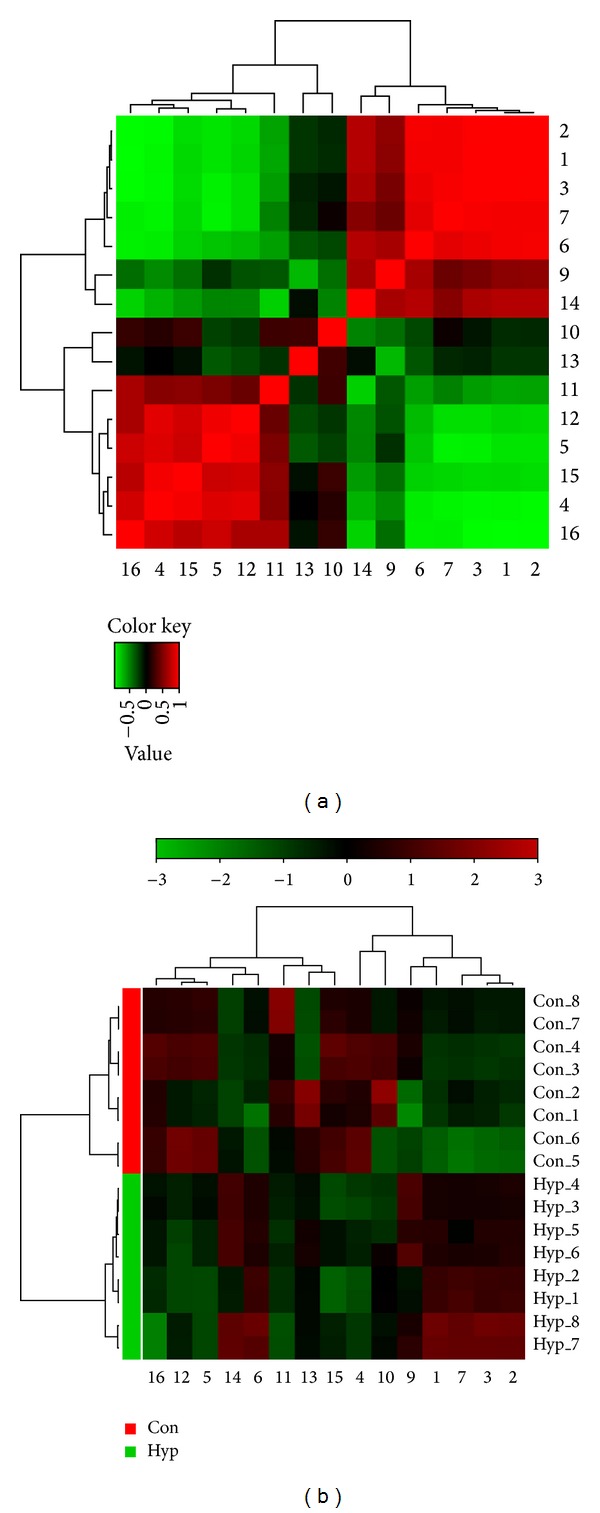
Correlation analysis (a) of the differential metabolites in control rats and diet-induced hyperlipidaemia rats. Heat map (b) for identified metabolites in control rats and diet-induced hyperlipidaemia rats. The color of each section is proportional to the significance of change of metabolites (red, upregulated; green, downregulated). Rows: samples; columns: metabolites. Numbers consist with Table 1.

To further understand the metabolic differences between diet-induced hyperlipidemia rats and control rats, identified metabolites were analyzed using correlation analysis and clustering heat maps. Correlation analysis showed the relation of differential metabolites. From the above plots, identified metabolites could be considered as being responsible for the separation between diet-induced hyperlipidemia rats and control rats and were therefore regarded as potential biomarkers. Heat map showed directly the variation of each metabolite, and the identified metabolites were visualized in a clustering heat map. Identified metabolites are showed in heat map, which shows the relative increase (red) or decrease (green) compared with control rats (Figure 4(a)). The hyperlipidemia model was capable of distinguishing diet-induced hyperlipidemia rats from control rats. The metabolites octadecanamide, oleamide, tryptophan, ursodeoxycholic acid, creatinine, 3-methyluridine, indole-3-carboxylic acid, and tryptophyl-tyrosine showed an increasing tendency in diet-induced hyperlipidemia rats. Although these metabolites showed similar tendencies to increase, some of the metabolites showed different increasing levels in diet-induced hyperlipidemia rats. In contrast, the metabolites citric acid, adenosine 2′,3′-cyclic phosphate, 3-O-methyldopa, proline, 1-methyladenosine, phenylalanine, and 5-methylcytosine showed a decreasing tendency in diet-induced hyperlipidemia rats. These results are consistent with the relative intensity and OPLS-DA loading plots.

Amino acids serve as substrates for protein synthesis, metabolic energy (oxidation through TCA cycle), or gluconeogenesis and ketogenesis [24]. Increased urinary tryptophan and tryptophyl-tyrosine and decreased urinary phenylalanine were observed in diet-induced hyperlipidemia rats. Tryptophan is essential amino acid which cannot be synthesized by the body. Tryptophan either participates in proteins or is broken down for energy and metabolic intermediates. Dopamine is an important neurotransmitter as 5-hydroxytryptamine, which is derived from tryptophan metabolism. 3-O-Methyldopa is one of the main biochemical markers for aromatic amino acid decarboxylase deficiency, which affects dopamine biosynthesis. An obvious decrease of 3-O-methyldopa was observed in diet-induced hyperlipidemia rats compared with control rats. Indole-3-carboxylic acid is the metabolite of tryptophan, the precursor of neurotransmitter 5-hydroxytryptamine. Level of indole-3-carboxylic acid was significantly increased in diet-induced hyperlipidemia rats compared with control rats. Phenylalanine is an essential amino acid and its hydroxylation by phenylalanine hydroxylase to tyrosine is the major metabolic pathway for phenylalanine. Significant fate of tyrosine is a conversion to the catecholamines, for example, dopamine, norepinephrine, and epinephrine [25]. Diet-induced hyperlipidemia associated with dysfunction of tryptophan and phenylalanine, which were identified as potential biomarkers for hyperlipidemia or atherosclerosis, has been demonstrated [9, 26]. Proline is the catabolite of peptide degradation by proline iminopeptidase and is a precursor of pyruvate. Pyruvate can be converted into acetyl-CoA, which is the main input for a series of reactions known as the TCA cycle. Decreases in levels of proline and citric acid were observed in diet-induced hyperlipidemia rats. Decrease in proline was related to the glutamate/P5C synthase pathway via inactivating P5C synthase or other enzymes involved [27]. The previous study demonstrated that urinary citric acid was significantly decreased in diet-induced hyperlipidemia [9]. These results indicated that amino acids metabolism and TCA cycle were disturbed in diet-induced hyperlipidemia rats.

Increased 3-methyluridine and decreased 1-methyladenosine and 5-methylcytosine were observed in diet-induced hyperlipidemia rats. It has been demonstrated that 3-methyluridine and 1-methyladenosine were significantly increased in the pathogenesis of Alzheimer's disease [28]. 1-Methyladenosine is one of the modified nucleosides; the level is elevated in urine of patients with malignant tumors. Examination of expression of 1-methyladenosine is expected to be useful for the histological diagnosis of intraocular tumors. However, the previous study did not report that 3-methyluridine, 1-methyladenosine, and 5-methylcytosine were identified as potential biomarkers in diet-induced hyperlipidemia rats.

The concentrations of octadecanamide and oleamide were higher in diet-induced hyperlipidemia rats than in control rats. Fatty acids are reported to be associated with atherosclerotic and inflammatory diseases because they are the major components of the cytoplasmic membrane and the precursor fatty acids for prostaglandins and leukotrienes [29]. Bile acids have many important physiological functions such as lipid absorption, cholesterol homeostasis, and generation of bile flow that help in the recirculation and excretion of exogenous and endogenous metabolites. Increased urinary ursodeoxycholic acid was observed in diet-induced hyperlipidemia rats compared with control rats. It has been demonstrated that increased ursodeoxycholic acid was associated with atherosclerosis and ursodeoxycholic acid was identified as a potential biomarker in atherosclerosis rats [26]. In addition, the previous study demonstrated that urinary cholesterol was significantly increased in diet-induced hyperlipidemia [12]. Creatinine is usually produced at a relatively constant rate by the human body. Although serum creatinine is a commonly used indicator of renal function, increased urinary creatinine is observed only when significant injury occurs in renal function. Therefore, an increased level of urinary creatinine observed in the diet-induced hyperlipidaemia rats might indicate renal injury caused by hyperlipidaemia.

## 4. Conclusions

Urinary metabolomics based on UPLC Q-TOF/HDMS, a novel MS^E^ data collection technique, and a multivariate statistical technique has been used to study diet-induced hyperlipidaemia in a rat model. The OPLS-DA score plot showed the complete distinction of diet-induced hyperlipidaemia rats and control rats. Furthermore, significant differences in the urinary levels of fatty acids, amino acids, nucleosides, and bile acids were observed in diet-induced hyperlipidaemia rats. These results demonstrated the perturbations of fatty acids metabolism, amino acid metabolism, and nucleosides metabolism of diet-induced hyperlipidaemia. This research also demonstrated that UPLC-MS-based metabolomics was a promising tool to find and identify potential biomarkers in diet-induced hyperlipidaemia rats.

## Figures and Tables

**Table 1:** 13 biomarkers of hyperlipidemia detected by UPLC Q-TOF/MS in negative ion mode in the 4th week.

Number	Mass	Metabolite	i-FIT^a^, elemental composition	Molecular weight	Formula	Trend^b^	Related pathway
1	284.2934	Octadecanamide	0.9, C_18_H_38_NO	283.4925	C_18_H_37_NO	↑**	Fatty acid metabolism
2	282.2779	Oleamide	1.2, C_18_H_36_NO	281.2718	C_18_H_35_NO	↑**	Fatty acid metabolism
3	188.0713	Tryptophan	0.9, C_11_H_10_N_1_O_2_	204.2252	C_11_H_12_N_2_O_2_	↑**	Amino acid metabolism
4	193.1235	Citric acid	0.7, C_6_H_9_O_7_	192.1243	C_6_H_8_O_7_	↓**	TCA cycle
5	330.0618	Adenosine 2′,3′-cyclic phosphate	1.0, C_10_H_13_N_5_O_6_P	329.2059	C_10_H_12_N_5_O_6_P	↓**	Purine metabolism
6	393.3002	Ursodeoxycholic acid	0.8, C_24_H_41_O_4_	392.5720	C_24_H_40_O_4_	↑**	Bile acid metabolism
7	114.0641	Creatinine	1.2, C_4_H_8_N_3_O	113.1179	C_4_H_7_N_3_O	↑**	Energy metabolism
8	281.0979	Ascorbalamic acid	0.9, C_9_H_17_N_2_O_8_	263.2014	C_9_H_13_NO_8_	↑**	Carbohydrate metabolism
9	259.0913	3-Methyluridine	1.2, C_10_H_15_N_2_O_6_	258.228	C_10_H_14_N_2_O_6_	↑**	Nucleoside metabolism
10	212.1025	3-O-Methyldopa	1.7, C_10_H_14_NO_4_	211.2145	C_10_H_13_NO_4_	↓**	Amino acid metabolism
11	162.1108	Proline	0.7, C_7_H_13_O_3_	115.1305	C_7_H_12_O_3_	↓**	Amino acid metabolism
12	282.1216	1-Methyladenosine	1.2, C_11_H_16_N_5_O_4_	281.2679	C_11_H_15_N_5_O_4_	↓**	Nucleoside metabolism
13	162.0538	Indole-3-carboxylic acid	0.8, C_9_H_8_NO_2_	161.1574	C_9_H_7_NO_2_	↑**	Amino acid metabolism
14	368.1594	Tryptophyl-tyrosine	1.2, C_20_H_22_N_3_O_4_	367.3984	C_20_H_21_N_3_O_4_	↑**	Amino acid metabolism
15	166.0708	Phenylalanine	0.9, C_9_H_12_NO_2_	165.1891	C_9_H_11_NO_2_	↓**	Amino acid metabolism
16	126.0643	5-Methylcytosine	1.3, C_5_H_8_N_3_O	125.1286	C_5_H_7_N_3_O	↓**	Nucleoside metabolism

^a^i-FIT; the i-FIT is the correctness of isotope patterns of elemental composition. The lower i-FIT normalized values mean high precision of the elemental composition; ^b^change trend of hyperlipidemia rats versus control rats. The potential biomarkers were labeled with (↓) downregulated and (↑) upregulated. *P < 0.05 and **P < 0.01.

## References

[1] Nicholson JK, Lindon JC, Holmes E (1999). ‘Metabonomics’: understanding the metabolic responses of living systems to pathophysiological stimuli via multivariate statistical analysis of biological NMR spectroscopic data. *Xenobiotica*.

[2] Nicholson JK, Connelly J, Lindon JC, Holmes E (2002). Metabonomics: a platform for studying drug toxicity and gene function. *Nature Reviews Drug Discovery*.

[3] Lindon JC, Holmes E, Bollard ME, Stanley EG, Nicholson JK (2004). Metabonomics technologies and their applications in physiological monitoring, drug safety assessment and disease diagnosis. *Biomarkers*.

[4] O’Keefe JH, Bell DSH (2007). Postprandial hyperglycemia/hyperlipidemia (postprandial dysmetabolism) is a cardiovascular risk factor. *The American Journal of Cardiology*.

[5] Esteve E, Ricart W, Fernández-Real JM (2005). Dyslipidemia and inflammation: an evolutionary conserved mechanism. *Clinical Nutrition*.

[6] Genest J, McPherson R, Frohlich J (2009). 2009 Canadian Cardiovascular Society/Canadian guidelines for the diagnosis and treatment of dyslipidemia and prevention of cardiovascular disease in the adult—2009 recommendations. *The Canadian Journal of Cardiology*.

[7] Adiels M, Olofsson S-O, Taskinen M-R, Borén J (2008). Overproduction of very low-density lipoproteins is the hallmark of the dyslipidemia in the metabolic syndrome. *Arteriosclerosis, Thrombosis, and Vascular Biology*.

[8] Wu DJ, Zhu BJ, Wang XD (2011). Metabonomics-based omics study and atherosclerosis. *Journal of Clinical Bioinformatics*.

[9] Liu F, Gan PP, Wu H, Woo WS, Ong ES, Li SFY (2012). A combination of metabolomics and metallomics studies of urine and serum from hypercholesterolaemic rats after berberine injection. *Analytical and Bioanalytical Chemistry*.

[10] Zhang X, Wu C, Wu H (2013). Anti-hyperlipidemic effects and potential mechanisms of action of the caffeoylquinic acid-rich *Pandanus tectorius* fruit extract in hamsters fed a high fat-diet. *PLoS ONE*.

[11] Zhang Q, Wang G-J, A J-Y (2009). Application of GC/MS-based metabonomic profiling in studying the lipid-regulating effects of *Ginkgo biloba* extract on diet-induced hyperlipidemia in rats. *Acta Pharmacologica Sinica*.

[12] Zhang Q, Wang G, A J (2010). Metabonomic profiling of diet-induced hyperlipidaemia in a rat model. *Biomarkers*.

[13] Morand J-PF, Macri J, Adeli K (2005). Proteomic profiling of hepatic endoplasmic reticulum-associated proteins in an animal model of insulin resistance and metabolic dyslipidemia. *The Journal of Biological Chemistry*.

[14] Zhao YY (2013). Metabolomics in chronic kidney disease. *Clinical Chimica Acta*.

[15] Wrona M, Mauriala T, Bateman KP, Mortishire-Smith RJ, O’Connor D (2005). ‘All-in-One’ analysis for metabolite identification using liquid chromatography/hybrid quadrupole time-of-flight mass spectrometry with collision energy switching. *Rapid Communications in Mass Spectrometry*.

[16] Zhao Y-Y, Liu J, Cheng X-L, Bai X, Lin R-C (2012). Urinary metabonomics study on biochemical changes in an experimental model of chronic renal failure by adenine based on UPLC Q-TOF/MS. *Clinica Chimica Acta*.

[17] Zhao YY, Shen X, Cheng XL, Wei F, Bai X, Lin RC (2012). Urinary metabonomics study on the protective effects of ergosta-4, 6, 8(14), 22-tetraen-3-one on chronic renal failure in rats using UPLC Q-TOF/MS and a novel MS^E^ data collection technique. *Process Biochemistry*.

[18] Zhao YY, Feng YL, Bai X, Tan XJ, Lin RC, Mei Q (2013). Ultra performance liquid chromatography-based metabonomic study of therapeutic effect of the surface layer of *Poria cocos* on adenine-induced chronic kidney disease provides new insight into anti-fibrosis mechanism. *PLoS ONE*.

[19] Zhao YY, Lei P, Chen DQ, Feng YL, Bai X (2013). Renal metabolic profiling of early renal injury and renoprotective effects of *Poria cocos*
epidermis using UPLC Q-TOF/HSMS/MS^E^. *Journal of Pharmaceutical and Biomedical Analysis*.

[20] Zhao YY, Li HT, Feng YL, Bai X, Lin RC (2013). Urinary metabonomic study of the surface layer of *Poria cocos* as an effective treatment for chronic renal injury in rats. *Journal of Ethnopharmacology*.

[21] Zhao YY, Zhang L, Long FY (2013). UPLC-Q-TOF/HSMS/MS^E^-based metabonomics for adenine-induced changes in metabolic profiles of rat faeces and intervention effects of ergosta-4, 6, 8(14), 22-tetraen-3-one. *Chemico-Biological Interactions*.

[22] Zhao Y-Y, Cheng X-L, Wei F (2013). Intrarenal metabolomic investigation of chronic kidney disease and its TGF-*β*1 mechanism in induced-adenine rats using UPLC Q-TOF/HSMS/MS^E^. *Journal of Proteome Research*.

[23] Zhao Y-Y, Cheng X-L, Wei F, Bai X, Lin R-C (2012). Application of faecal metabonomics on an experimental model of tubulointerstitial fibrosis by ultra performance liquid chromatography/high-sensitivity mass spectrometry with MS^E^ data collection technique. *Biomarkers*.

[24] Lopansri BK, Anstey NM, Stoddard GJ (2006). Elevated plasma phenylalanine in severe malaria and implications for pathophysiology of neurological complications. *Infection and Immunity*.

[25] Fernstrom JD, Fernstrom MH (2007). Tyrosine, phenylalanine, and catecholamine synthesis and function in the brain. *The Journal of Nutrition*.

[26] Zhang F, Jia Z, Gao P (2009). Metabonomics study of atherosclerosis rats by ultra fast liquid chromatography coupled with ion trap-time of flight mass spectrometry. *Talanta*.

[27] Watford M (2008). Glutamine metabolism and function in relation to proline synthesis and the safety of glutamine and proline supplementation. *The Journal of Nutrition*.

[28] Lee SH, Kim I, Chung BC (2007). Increased urinary level of oxidized nucleosides in patients with mild-to-moderate Alzheimer’s disease. *Clinical Biochemistry*.

[29] Dyerberg J (1986). Linolenate-derived polyunsaturated fatty acids and prevention of atherosclerosis. *Nutrition reviews*.

